# Early warning signals of recovery in complex systems

**DOI:** 10.1038/s41467-019-09684-y

**Published:** 2019-04-11

**Authors:** Christopher F. Clements, Michael A. McCarthy, Julia L. Blanchard

**Affiliations:** 1 School of Biological Sciences, University of Bristol, Bristol, BS8 1TQ UK; 2 School of BioSciences, University of Melbourne, Parkville, Melbourne, 3010 Australia; 3 Institute for Marine and Antarctic Studies and Centre for Marine Socioecology, University of Tasmania, Hobart, TAS 7001 Tasmania Australia

## Abstract

Early warning signals (EWSs) offer the hope that patterns observed in data can predict the future states of ecological systems. While a large body of research identifies such signals prior to the collapse of populations, the prediction that such signals should also be present before a system’s recovery has thus far been overlooked. We assess whether EWSs are present prior to the recovery of overexploited marine systems using a trait-based ecological model and analysis of real-world fisheries data. We show that both abundance and trait-based signals are independently detectable prior to the recovery of stocks, but that combining these two signals provides the best predictions of recovery. This work suggests that the efficacy of conservation interventions aimed at restoring systems which have collapsed may be predicted prior to the recovery of the system, with direct relevance for conservation planning and policy.

## Abstract (web summary)

While several studies have documented early warning signals of population collapse, the use of such signals as indicators of population recovery has not been investigated. Here the authors use models and empirical fisheries data to show that there are statistical indicators preceding recovery of cod populations.

## Introduction

In the 1990s one of the most iconic regime shifts occurred when cod and other groundfish stocks in the Northwest Atlantic collapsed and led to a moratorium on fishing that has lasted 30 years, with few signs that the system is recovering^1^. This failure to recover is thought to be an example of the phenomenon of hysteresis, where a system can display multiple stable states under the same or similar environmental conditions^2–5^. In practical terms this means that a system’s state can be a function not only of the current environmental conditions but also of its previous state^2–5^. Classic examples of this come from freshwater lakes, characterized by states with either clear-water and macrophytes, or turbid-water and plankton;^4^ reverting a system from a turbid to clear-water state is demonstrably difficult^6^. The transition between these two states, which is often abrupt, is referred to as a tipping point, and here we will use this to describe the point at which a system rapidly changes either by passing through a bifurcation point in the classic mathematical sense, or via a period of strong non-linearity^7^. The need to try and avoid these abrupt transitions between alternative states, typified by the collapse of populations which then exhibit hysteresis, has helped drive the development of so called generic early warning signals to predict the collapse of ecological systems (Early Warning Signals of Collapse, henceforth EWSCs) in the face of global environmental change^8^.

Thus far EWSCs have largely been derived from the theory of critical slowing down (CSD), whereby a system exhibits a decreasing ability to return to its previous state after a perturbation, driven by a decline in its resilience in the region of a tipping point^7–9^. In practical terms, this decline in the resilience of a system can be inferred from changes in readily measurable statistics of an abundance time series; as a system approaches a collapse the autocorrelation should increase, as should variance and a suite of other statistical moments^5,10^. Such generic EWSCs have been shown to be present prior to the collapse of a range of non-biological and biological systems^10–17^. However, recent work has shown that such methods are highly susceptible to poor data quality and the low signal to noise ratio of biological systems^18–21^, driving the development of trait-based EWSCs, where shifts in the mean and distribution of a fitness related phenotypic trait such as body size can help predict the collapse of populations and communities^11,12,22^. Such trait-based methods have been proposed as being more robust to the ubiquitous incomplete sampling which occurs when monitoring wild populations^12,18^, as they track shifts in the mean of a sample, rather than assuming the sample directly reflects the true state of the system (as with population count data)^5^. These abundance and trait-based tools give hope that the collapses of populations, communities, and ecosystems may be predicted prior to their occurrence. However, one overlooked fact is that such signals are predicted to be present regardless of the direction from which the tipping point is approached – i.e. warning signals are expected to be present before both the collapse and the recovery of complex systems^7^. No work has assessed whether these predicted signals of recovery are seen in realistic population dynamic data, or what role any potential signals may play in the management of populations and communities in the face of continued anthropogenic forcing of the global biosphere. Moreover, it is not known whether previously developed trait-based warning signals (which are not derived from bifurcation theory) would also be present prior to the recovery of a system.

While the collapse of populations and communities is of primary concern for conservation biologists given global declines in biodiversity, early warning signals prior to the recovery of a system (henceforth EWSRs) have the potential to play a critical and unique role in assessing the effects of conservation interventions and management decisions. Measuring the effectiveness of a conservation intervention is critical to ensure limited resources are not wasted on a management strategy which is having no positive impact on the intended target. Moreover, the risk of further collapse, the extinction of a population, or loss of function of an ecosystem, must be minimised by reducing the time the system spends in a collapsed phase. However, predicting the efficacy of management strategies is hampered by the complexity of biological systems, and thus developing generic EWSRs could offer many of the same advantages which drove the development of classic EWSCs; they may act as generalisable pattern-to-process methods, requiring relatively little data on a system to predict its future state^5^. This would be of particular interest for the management of complex communities and ecosystems, where monitoring and understanding the interactions between large numbers of populations simultaneously may be impractical, but where the assessment of key species (either of commercial interest, or which are known to play important roles in the functioning of the system) may not only be feasible but already being undertaken. This points to one of the key advantages of EWSRs over classic EWSCs: the utility of EWSCs in predicting the collapse of real-world systems is often hampered by the quality and availability of the data^18,19^, as typically populations are monitored when they are already highly threatened or declining, and thus warning signals are less relevant. However, populations which have already collapsed are likely to be carefully monitored, especially in high economic sectors such as fisheries, and thus developing generic methods to predict the recovery of such systems as management interventions are enacted takes advantage of this, removing a key limitation of traditional EWSCs methods^18,19^. Such generic signals may be of particular utility for developing nations, where complex and expensive mechanistic models of fisheries may be absent, but measures of a system’s health such as the size of individuals in the population may be collected from fish markets^11,23^. As such, EWSR have the potential to add to the toolbox of methods to help inform conservation funding and prioritisation in a wide variety of systems.

Here we use a previously developed multi-species size spectrum model of the North Sea to simulate the recovery of an over-harvested system when fishing pressure is released^24^. This model realistically predicts the effects of harvesting and interspecific interactions on the abundance and trait dynamics of this marine fish community^24^, while allowing multiple outcomes (populations that recover vs. those that do not) to be readily generated. We use in silico experiments to first implement the recorded historic fishing pressures between 1967 and 2010 across all species, leading to large declines in the biomass of many of the species, and then subsequently reduce fishing pressure to zero at various rates. Given the wide economic importance of Atlantic cod (*Gadus morhua*) and issues surrounding the collapse and recovery of cod stocks, in our analyses we focus on the North Sea where cod has declined for much of the past four decades. Reductions in fishing mortality rates have led to some signs of recovery in recent years, however according to stock assessment and biological reference levels the North Sea cod stocks still remain “outside safe biological limits”. Additional methods to assess whether this system is likely to recover further would help provide a greater evidence base for fisheries management. Here we assess, whether, when fishing pressures on the system are relaxed, abundance and trait-based warning signals predict non-linear transitions of recovering of cod stocks. We show that both abundance and trait-based signals are independently detectable significantly prior to the recovery of cod, but that combining these two independent measures of the stability of a system provides the best predictions of recovery. To supplement this simulation-based approach we also analyse two real-world cod stock survey time series from the International Council for the Exploration of the Sea (ICES). One of these cod stocks (in the North Sea) is purportedly recovering from a period of over exploitation and displays EWSRs. The second stock, from the Western Baltic, is in contrast not thought to be recovering, a supposition supported by our analysis where it shows no EWSRs.

## Results

### Simulated data

**Fig. 1:**
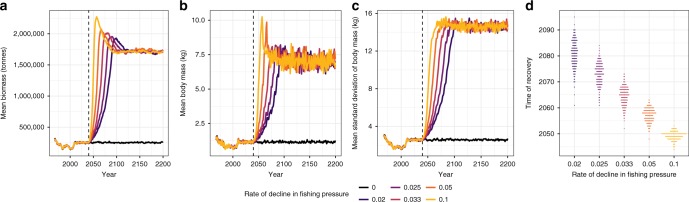
Projected cod population dynamics and recovery times under various rates of decline in fishing pressures. The model predicts significant increases in (**a**) mean biomass, (**b**) mean body size, (**c**) *σ* size as fishing pressures decline, with recovery times (**d**) changing approximately linearly with the rate of decline in fishing pressure. Each line represents the mean of 300 simulations

**Fig. 2:**
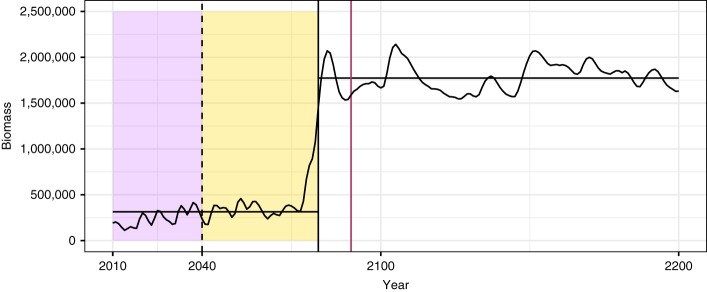
Example time series of cod recovery. The recovery point (vertical solid black line) was calculated by fitting a piecewise constant model with a single break point (horizontal black lines). The time at which fishing pressure begins to decrease (2040, vertical dashed black line), and the time at which fishing pressure declines to zero (vertical maroon line) are also shown. The purple box highlights the training data period used in the warning signal calculation, while the yellow box highlights the period over which warning signals of recovery were searched for (after fishing pressure has begun to decline, but prior to the population recovering)

Reductions in fishing pressures altered the trajectories of cod population recoveries, with the recovery of populations often lagging behind the change in fishing pressures and thus the populations displaying non-linear dynamics (Figs. 1 and 2). On average the recovering populations displayed over-compensatory dynamics, especially where the rate of decline in fishing pressure was rapid, leading to a dramatic increase and then decline in the biomass and mean body size as the pressure on the system was released (Fig. 1).

**Fig. 3:**
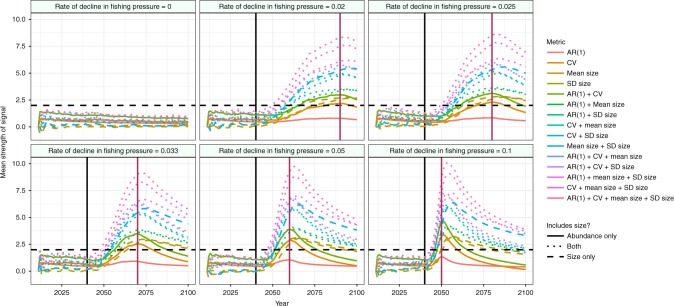
Mean trends in the strength of EWSR compromised of trait and abundance signals across 300 simulations of each rate of decline in fishing pressure. Those metrics that include size and abundance-based measures of stability (dotted lines) had significantly stronger signals of recovery than those based on either size (dashed lines) or abundance (solid lines) alone. Horizontal black lines indicate a 2*σ* threshold where a warning signal is considered as present under the method proposed by Drake & Griffen^14^. Vertical black lines indicate the point in time where fishing pressures begin to decline, vertical red lines show the point at which fishing pressures had fallen to 0

As the pressures on the system declined, the abundance and size-based metrics increased compared to both their historic base line, and the control populations (Fig. 3). Of the 15 metrics tested, those which included some measure of trait dynamics alongside abundance-based measures of stability had the strongest signals of recovery, with the strength of the signal typically peaking simultaneously with the cessation of fishing (Fig. 3). The maximum strength of the signal observed was proportional to the rate at which fishing pressures declined.

**Fig. 4:**
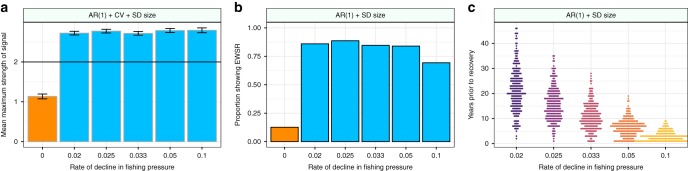
Efficacy of single warning signals in predicting the recovery of cod populations. **a** Metric with the greatest difference between the signal strength in the non-collapse and collapse time series, the greatest mean strength across all rates of decline, and the highest mean signal strength in any single rate of change (AR(1) + CV + SD size) of EWSR between the start of the decline in fishing pressure (2040) and the recovery time of each time series. Error bars show 1 SE. **b** Metric with the greatest difference between the proportion of time series showing false positive signals at a 2 sigma threshold (orange bars) and proportion of time series showing true positive signals (blue bars) – AR(1) + SD size. **c** Distribution in time of EWSR at a 2 sigma threshold for the metric AR(1) + SD size

Critical to indicators being useful in real world scenarios is their ability to predict approaching recoveries before they occur. When the maximum strength of signals between 2040 (the time when fishing pressures began to be reduced) and the recovery point of each population (as calculated by piecewise regression analysis, Fig. 2) were compared to those populations which didn’t recover there were clear and marked differences between the treatments. Specifically, those populations which recovered showed on average stronger signals than those that didn’t (Fig. 4a, Supplementary Fig. 4). For the metric which had the greatest difference in mean strength between the recovery and non-recovery populations (a combination of first order autoregressive coefficient, coefficient of variation, and the standard deviation of size – AR(1) + CV + SD size) the mean signal strength between 2040 and the recovery time of each population was significantly greater than the 2*σ* threshold proposed by Drake & Griffen^14^ (Fig. 4a). In those simulations which did not recover the mean maximum signal strength remained below this threshold level over the period of time between 2040 and 2090 (Fig. 4a).

This marked difference in the mean metric strength between the non-recovery and recovery populations generated a high number of true positive (TP) warning signals of recovery (Fig. 4b, Supplementary Fig. 5). At the slowest rate of decline in fishing pressure the metric AR(1) + SD size displayed TP EWSR in over 86% of simulations, and false positive (FP) signals in only 13% of the non-recovery populations. These EWSRs were detectable up to 46 years prior to the recovery of some populations, although the majority of such signals occurred closer to the recovery point than this (Fig. 4c).

**Fig. 5:**
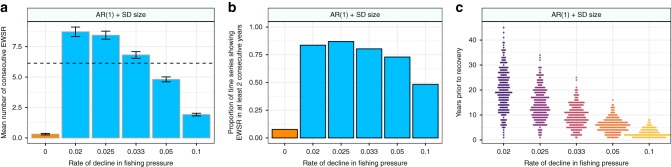
Efficacy of consecutive warning signals in predicting the recovery of cod populations. **a** Mean maximum number of consecutive EWSR for the metric with the highest mean maximum number across all the recovery simulations (blue bars, AR(1) + SD size) at a 2*σ* threshold. While those populations which did not recover did show some EWSR (orange bar), they typically did not show them in consecutive years, whereas those populations that did recover showed signals at a 2*σ* threshold in – on average – 6 consecutive years across the recovery treatments (horizontal black line). **b** Proportion of populations showing EWSR in at least 2 consecutive years at a 2*σ* threshold. Error bars show 1 SE. **c** Distribution in time of EWSR based on 2 consecutive signals at a 2*σ* threshold for the metric with the biggest difference in the number of consecutive signals between recovery and non-recovery treatments (AR(1) + SD size, Fig. 5). EWSR were detectable – where change in the pressures exerted upon a system is slow – up to 43 years prior to the recovery of the populations

Previous work has suggested that in noisy real-world time series multiple consecutive signals may provide more reliable predictors of an approaching tipping point, as the system becomes increasingly unstable and thus should exhibit increasingly frequent warning signals^11^. This was supported by the mean number of consecutive EWSRs generated in the recovery populations when compared to those which do not recover, where populations with the slowest rate of change in fishing pressure on average produced warning signals at a 2*σ* threshold in 8 consecutive years prior to their recovery (Fig. 5a, Supplementary Fig. 6). If a consecutive signal approach was implemented, and thus an EWSR was considered present when the metric crosses a 2*σ* threshold for 2 or more consecutive years, TP EWSRs were still produced in a high proportion of simulations (up to 83%), while FP signals were present in only 7% of the non-recovery simulations (Fig. 5b). Consecutive signals were not detectable as far in advance of the recovery point as the single signal approach (Figs. 4c and 5c), however they were none-the-less present up to 45 years prior to recovery in the slowest rate treatment (Fig. 5c).

**Fig. 6:**
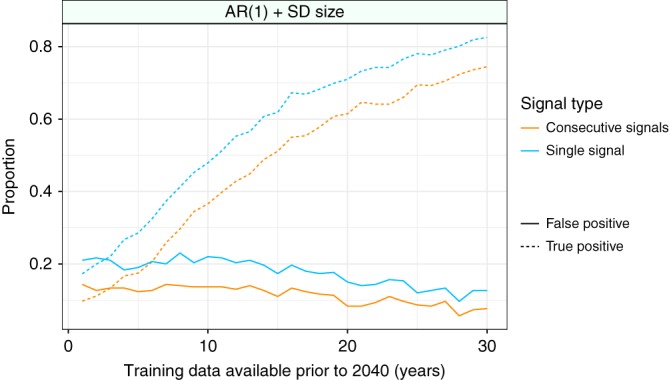
The effects of the amount of training data available on the proportion of false positive and true positive warning signals prior to the recovery of cod stocks for AR(1) + SD size. As the amount of training data available increased so too did the predictive accuracy of the method, with a high proportion of true positive (dashed lines) and low proportion of false positive (sold lines) signals when 30 years of training data are available. The consecutive signal approach (orange lines) provided consistently more cautious predictions (lower proportion of false positive and true positive signals than the single signal approach, blue lines)

Increasing the amount of training data available prior to the cessation of fishing increased the proportion of TP, and decreased the number of FP, signals (Fig. 6, Supplementary Fig. 7). Both the single and consecutive signal approach showed very similar trends as the amount of training data increased, with the single signal approach showing both higher TP and FP signals than the consecutive signal approach.

**Fig. 7:**
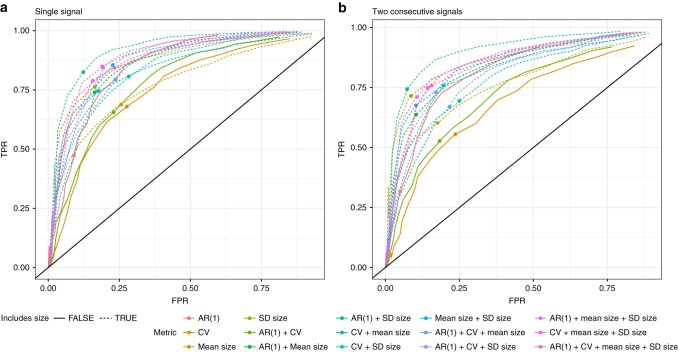
Receiver Operator Characteristics (ROC) for the 15 early warning signals metrics. Where (**a**) a single signal over the threshold was considered a warning signal of recovery, and (**b**) when two consecutive signals were considered a warning signal of recovery across a range of threshold sigma values between 0.01 and 6. FPR = False Positive Ratio, TPR = True Positive Ratio. Points show the location on the curve corresponding to a 2-sigma threshold

The 2*σ* threshold proposed for determining the presence of warning signals^14^ performed well in comparison to other threshold values, typically producing low FP ratios and high TP ratios when either a single signal, or consecutive signal approach was taken (Fig. 7). Of the 15 metrics tested AR(1) + SD size produced the best TP to FP ratio across the thresholds tested (Fig. 7).

### Fisheries survey data

**Fig. 8:**
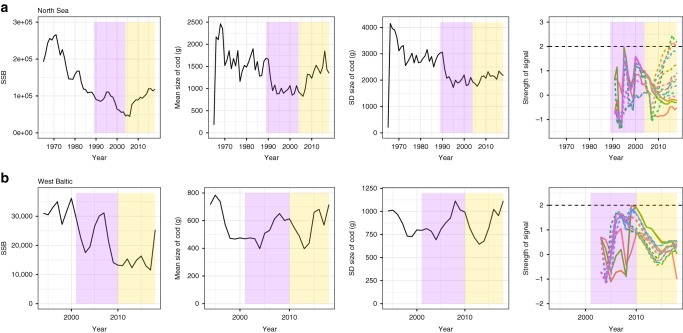
ICES cod survey data for the North Sea and Western Baltic Sea. Panels show spawning stock biomass (SSB), mean size, and SD size dynamics of the (**a**) North Sea and (**b**) West Baltic Sea cod stocks, as well as the dynamics of the warning signals of recovery. The purple box highlights the training data period used in the warning signal calculation, while the yellow box highlights the period over which warning signals of recovery were searched for (after fishing pressure has begun to decline, but prior to the population recovering). Warning signals of recovery are considered as present when the lines exceed a 2*σ* threshold (horizontal black dashed lines). Solid lines show metrics which do not include some measure of size, dashed lines indicate a metric includes either mean size or SD size or both

The North Sea cod stock has shown significant historic downward trend, with corresponding declines in both the mean size and SD body size of the population (Fig. 8a). Although the SSB has increased since 2006 it remains below historic levels. This increase has occurred concurrently with increases in the mean size, and (although to a lesser extent) SD size of individuals in the population (Fig. 8a). While the SSB is increasing slowly this population is generally considered to be showing signs of recovery^25^ and our analysis corroborates this, with signals detectable in multiple consecutive years from 2011 onwards in 3 of the 15 metrics, all of which included some measure of size (Fig. 8a).

The Western Baltic Sea’s population dynamics are less clear, with the SSB, mean size, and SD size showing significant interannual fluctuations, and no significant increases in any of these three measures (Fig. 8b). The metric dynamics also show significant variation, although the 2*σ* threshold is not crossed during either the training or assessment periods (Fig. 8b). These results seem to support the published view that the Western Baltic stock is not recovering^26^.

## Discussion

While work on EWSCs has grown rapidly^5^, the theoretical prediction that warning signs precede the recovery of complex systems has been overlooked. However, given the current losses of biodiversity and degradation of ecosystem structures and functions worldwide, predicting whether a system might recover given a conservation intervention may provide a key quantitative tool. We suggest that pattern-to-process methods based on recently developed theory may offer one potential solution to this issue, providing generalizable signals of recovery which require relatively little data or understanding of the underlying structure of a system to calculate. Such signals may be of particular use for developing nations, where a deep understanding of the complexities of their exploited ecosystems may be lacking.

We show that, prior to the simulated recoveries of cod, abundance-based and trait-based EWSRs are independently detectable, but that the most reliable signals occur when these two signal types are combined (Figs. 3–5). Moreover, these signals are detectable in a real-world system which is thought to be recovering (Fig. 8a) and are absent from one which is not (Fig. 8b). This finding supports previous work on the collapse of biological systems which has suggested that combining multiple signals (both trait and abundance) into a single metric of risk reduces the chances of FP signals^7,11,12,14^, which are often driven by chance fluctuations in the data caused by stochasticity or poor data quality^18^. In marine systems such as those simulated here including trait shifts as a warning signal of either collapse or recovery is particularly relevant, as populations are highly size structured^27^. That being said, the use of shifts in body size as a measure of increasing or decreasing stability in non-marine systems also has significant theoretical support, as many organism’s survival, fecundity, and response to environmental conditions and density dependency are determined by body size^28–30^. This in turn affects demographic rates and a population’s ability to withstand future pressures exerted upon it, or recover from past stressors^30–33^.

However, as with EWSCs, a key issue with the utility of EWSRs is their detectability. Historic work in the EWSCs literature shied away from providing quantitative decision-making frameworks, concentrating instead on proof-of-concept correlative studies analyzing data on systems which were known to have collapsed e.g.^16^, meaning that not only were such methods impractical for predicting real-world collapses, but were also susceptible to the ‘prosecutors fallacy’^34^. Consequently, for EWSCs/EWSRs to be useful the literature must strive to move on to frameworks through which clear signals can be achieved^5^. One such approach was developed by Drake & Griffen^14^, whereby an EWSC was determined to be present when the value of a metric at a given time point exceeded its running mean by more than two standard deviations – effectively a 97.5% confidence level^12^. Employing such a method for the detection of EWSRs appears to have merit, as while FP signals do occur, they are proportionally low when compared to the TP signals (Figs. 4b and 7).

However, the simulation data we analyze here represents a best-case scenario. Real world data are considerably more noisy, driven by incomplete sampling and the movement of organisms^12,18^. In practical terms, noisy data may lead to increased FP and false negative (FN) signals, meaning a system’s future trajectory may be incorrectly identified, leading to wasted resources or worse the further collapse or total extirpation of the system. One option would be to alter the threshold *σ* value, attempting to balance the economic, political, and ecological costs of a FP or FN signal. However, there is little objectivity in choosing a new threshold. Recognized issues such as the Romeo effect^35^, where a population or species is assumed to be lost or unsavable leading to the removal of funding and its subsequent committal to extinction when in reality it could have been saved, is a clear example of both the dangers of misidentifications of signals, and potential benefits of using reliable quantitative methods to inform conservation planning. In reality the 2*σ* threshold previously suggested appears to perform well compared to other threshold values (Fig. 7), and thus arbitrarily altering this value may offer little benefit.

An alternative method to solve this issue of reliability in the face of noisy data may be to use signals in consecutive years as a measure of the loss of stability of a system^11^ (Fig. 5). Given that the majority of FP signals are generated from chance fluctuations in the data, such an approach has merit (Fig. 5b), as multiple consecutive signals suggest that a system has not only moved beyond its historic baseline, but that it is continuing to do so in response to some external pressure. Our results suggest that two consecutive signals can predict an approaching recovery of cod stocks, and that using this approach significantly decreases the FP signal rate (Figs. 5 and 6).

To assess whether such signals may be detectable in real-world recovery cases we analyse two systems, one which is thought to be recovering and one which is not, to assess whether EWSRs are present (Fig. 8). Using the methods developed in the simulation study we show that multiple consecutive signals are detectable in the ‘recovering’ system, while no signals are detectable in the ‘not recovering’ system (Fig. 8). Based on these results we would predict that the North Sea should show continued future recovery over the next decades, assuming that pressures on the system are not once again increased, although the strength of this signal of recovery only just passed the 2*σ* threshold in 3 of the 15 metrics (Fig. 8a). It should be noted that the time series used for training the methods in the real-world data analysis are shorter than the best-case scenario presented in the simulation study (15 years for the North Sea, and 9 years for the Western Baltic), and thus are likely to be more susceptible to variation in the data (Fig. 6). The choice of window size is also likely to alter these results, an issue with many different warning signal approaches e.g. ^10^, and thus while such signals appear to support the results of the modelling, they should not be treated as conclusive proof that such signals exist in real-world systems. In particular, the absence of recovery signals does not prove that no recovery is occurring in the Western Baltic, as recovery in the immediate future cannot be ruled out.

As with all predictive methods the quality of the data available will drive the reliability of EWSRs^18^, and a critical component of data quality is the length of the time series available. Our results demonstrate that with data covering a collapse period of a similar length to that observed in real world cod stocks (~30 years)^1^ reliable signals of recovery can be generated (Fig. 6 and Supplementary Fig. 7). However, this might be considered a best-case scenario, as in many instances’ conservation data are limited both temporally and spatially (Fig. 8b). While the abundance-trait methods we propose perform well when the amount of training data for the methods is high (>20 years), when the amount of training data is low the methods perform poorly (Fig. 6), a finding in line with previously published work on EWSC^11,12^. The requirement for relatively long (20 year) data has and continues to be a significant issue for the use of classic EWSC^5,18^. However, EWSR may not be limited in the same way, as monitoring of a system often occurs when degradation of the system is observed. That being said the analysis of short time series should still be treated with caution.

The results presented here must be set in the context of previous work which has highlighted the limitations of CSD based methods for predicting tipping points^20^. Boerlijst et al.^20^ highlighted the inability of CSD based methods to predict the collapse of a number of ecological models, suggesting that to correctly predict the collapse of a system a detailed knowledge of the mathematical structure of any potential bifurcation is needed. Chief amongst these concerns may be that CSD will only occur in the direction of the dominant eigenvector, which, in their example, may occur in only the juveniles of a population, which may not be monitored (for example in marine fish stocks)^20^. In our analyses we assess the presence of CSD and trait-based indicators only in individuals above 10 g, typically the minimum sized individual monitored during marine stock surveys^24^. Thus we are able to show that in this system weak CSD based signals are present, as well as strong ones in the trait-based metrics. However, although subsequent work has shown that in fact a mathematical bifurcation need not occur to produce CSD based warning signals^7^, the fact remains that some biological systems may not exhibit CSD and its associated warning signals prior to their collapse^21^. Trait-based warning signals, which are not shackled by bifurcation theory^5^, may well solve many of these issues, but only in systems where body-size is inherently linked to the demography of a population, as in the one we present here.

In conclusion, we show that the recovery of complex systems can be predicted by early warning signals based on both the statistical moments of biomass data and the dynamics of body size, but that combining such signals into a single metric produces the most reliable signals of recovery. Such abundance/biomass and trait-based metrics are widely generalizable, as they make few assumptions about the underlying structure and function of the system, and as such could provide a key tool in the restoration of degraded systems.

## Methods

### Multispecies size spectrum model

Interactions between species in fisheries play a critical part in the dynamics of the system as a whole, with interspecific and intraspecific interactions driven by the size of individuals in the populations, and thus their ability to prey or be preyed upon^24^. Here we employed a previously developed multispecies dynamic size spectrum model of the North Sea with 12 interacting species and a background resource community, which has been shown to provide realistic size spectra and population dynamics^24^. This is a dynamic continuous time and size model based on the McKendrick von Foerster equations which is discretized for numerical approximation using finite upwind differencing methods^24^. The timestep was discretized at 0.1 years to ensure the integration routine was satisfactory, and size was discretized to 100 size classes. We analyzed and present yearly outputs from the model to reflect the yearly monitoring of fisheries stocks. We then focused on assessing the recovery dynamics of replicate cod populations for EWSRs. Although the underlying model is deterministic (and is available in the R package ‘mizer’^36^), as in Blanchard et al.^24^ we use the stochastic version that includes a log-normal error term on the recruitment for all 12 fish species. The model outputs included abundance and biomass of each species and their body size distributions through time, however in the analyses we considered only the dynamics of cod. When calculating abundance, biomass, and the change in mean body size of a population through time we only considered individuals larger than 10 g in our calculations. This was to ensure that the observed dynamics of the system match those which could be observed when surveying this system in the real world, where 10 g is typically the minimum size caught during fish surveys^24^.

### Change in fishing pressures

In line with Blanchard et al.^24^ a burn-in period of 300 years (between 1667 and 1967AD) was used to ensure the model reached equilibrium before carrying out time-varying fishing simulations. After this period, fishing was implemented at recorded historic levels for all of the 12-species in the model between 1967 and 2010 (Supplementary Fig. 1)^24^. To provide an example of an overexploited marine system, we held fishing pressures constant between 2010 and 2040 (at 2010 rates), producing a 30 year ‘collapse period’ where cod do not recover, similar to that observed in real-world systems^1^. Subsequently, we simulated a range of post collapse scenarios (2040 onwards), where fishing pressures either remain constant (producing a persistent collapsed community) or decreased linearly across all species until fishing pressures reached 0, which allowed the system to recover from collapse (Supplementary Fig. 1). Because the rate at which pressures on a system changes can alter the prevalence of EWSCs^37^, we simulated five different rates of decline in the strength of fishing pressure (2%, 2.25%, 3.3%, 5%, and 10% per year declines in the 2010 level of fishing pressure, equivalent to declining to no fishing linearly over a 10, 20, 30, 40, or 50 year period). Each of these six treatments (5 recovery and one control where fishing pressures were held at 2010 levels) were simulated 300 times over a period from 1667 to 2200, giving a total of 1800 simulations, of which 1500 recover and 300 do not (Fig. 1, Supplementary Figs. 1 and 2). To avoid any artefacts of the data generation process, these 1800 simulations were split into three groups of equal size containing 100 simulations of each of the 6 rates of change in fishing pressure, and the stochastic simulations were initialized with the random number generators set at differing start points. We then focus our search for early warning signals solely on the simulated dynamics of cod (Fig. 1), presenting the full community dynamics in the supplementary information (Supplementary Figs. 1, 2 and 3).

### Calculating recovery times and EWSRs

For the EWSRs analysis, we focused solely on the simulated dynamics of cod (Fig. 1). For each of the 1500 simulated cod populations where fishing pressures declined over time, we calculated the point at which a population was considered to have recovered, estimated by fitting piecewise constant models with a single break point (Fig. 2)^38^. We then assessed whether EWSR occurred in the simulated cod populations. For populations which recovered we looked for signals between 2040 (when fishing pressures began to be reduced) and the estimated recovery point of the population, thus any signals detected in this period could be considered to predict the recovery of the population as they occurred prior to its observed recovery (Fig. 2). For those populations which were subjected to constant fishing pressures we assessed whether any false positive (FP) EWSRs occurred over a 50-year period between 2040 and 2090 (equivalent to the longest time series where populations recover, rate of change = 0.02).

We assessed the presence of two types of EWSRs: (1) signals calculated from biomass time series which are based on the theory of CSD, and (2) recently developed trait-based early warning signals which assess trends in the body size of individuals in a population^5,11,12^.

CSD suggests that a range of summary statistics calculated from the abundance or biomass time series of a population will show strong trends as the system approaches a tipping point^10^. These statistics include the coefficient of variation (*CV*), first order autoregressive coefficient (AR(1)), autocorrelation at first lag (ACF1), return rate (rr), and density ratio (DR). Strong trends in these in the direction expected by theory have preceded transitions between alternate states in a range of systems^10,14,16,18^. However, many of these metrics show strong collinearity^18^, so here we assessed the suitability of one noise based metric (CV) and one memory based statistic (AR(1)) to predict the recovery of populations, as the combination of these two types of signal has been proposed as a robust method for predicting the future state of a system^7^. CSD theory suggests that both CV and AR(1) should increase as a system approaches a tipping point^10^.

An alternative suite of previously suggested warning signals are trait-based measures of stability, such as shifts in the mean body size or standard deviation of body size of a population^11,12^. Because body size determines the survival and fecundity of many species^28–30^, shifts in the traits of a population offer an excellent alternative measure of stability to abundance-based measures derived from CSD^12^. Trait-based signals may be particularly relevant to marine systems, where not only do pressures such as fishing alter the size distribution of harvested populations, with cascading effects to the stability of the system^39^, but body-size also determines an individual’s position in the foodweb^24^. Previous work on overharvested marine systems has shown that declines in the mean size (mean size) and standard deviation of size (SD size) of a population predicts its collapse^11^. Because of this we consider increases in mean size and the SD size of the simulated cod populations as warning signals of their recovery.

Thus, in total we assess the efficacy of four population-level metrics to predict the recovery of the overharvested cod stocks in our model, two based on trait dynamics (mean size, SD size), and two based on biomass dynamics (AR(1), CV). These four metrics were assessed both independently, and by combining multiple indicators into a single metric of risk by summing them at each time point, as in Drake and Griffen^14^. Thus in total we tested 15 different metrics, composed of every unique combination of one to four indicators.

In line with previous work^11,12,14^ we assessed the presence of warning signals by normalizing each indicator (CV, AR(1), mean size, SD size) independently by subtracting the running average of that indicator from the value of that indicator at time *t*, and dividing it by the running standard deviation. Thus, each statistic at time *t*$(\hat w_t)$ was calculated as

1 $$\hat w_t = \frac{{w_t - \bar w_{1:t}}}{{\mathrm{sd}\left( {w_{1:t}} \right)}}$$

where $\bar w_{1:t}$ is the mean of a statistic from times 1 to t, and sd(*w*_1:*t*_) is the standard deviation over the same period, where *t* ≥ 2. Where multiple indicators were combined into a single metric, the values for each indicator to be included were summed at each time point, giving a composite EWSR^12,14^. To minimize the effects of noisy data generating erroneous warning signals, we take an approach similar to that of Dakos et al.^10^ by using a 30 year training period for the model (from 2010 – the start of the collapse period when fishing pressures are fixed – to 2039 – the year prior to fishing pressures being reduced, Fig. 2). Data from this training period were included in the analysis, but any warning signals generated were disregarded. Our analysis thus assessed whether the release of fishing pressures generated EWSRs using training data from a lengthy collapse period. We also assessed the sensitivity of our results to the length of this training data.

We present several analyses of these normalized indicators. First, we present the $\hat w_t$ values at each time point to show how the indicators change as the strength of fishing pressures is decreased (Fig. 3). Note that this does not indicate whether these signals precede the calculated recovery points but rather displays their behavior as the system recovers and stabilizes. Second, we show the mean value of $\hat w_t$ (averaged across replication populations in each treatment) between the start of fishing pressure decline and the recovery of the populations for the best composite metric (Fig. 4a). Thirdly, we show the proportion of populations which show warning signals of recovery using the method described by Drake and Griffen^14^ where a warning signal is considered to be present when $\hat w_t$ > 2 (i.e. when the value of a normalized indicator exceeded its running mean by two standard deviations) (Fig. 4b), and the distribution of these warning signals prior to the recovery of the simulated populations (Fig. 4c). Fourthly, we show the efficacy of using signals at a 2*σ* threshold in two consecutive years (previously identified as a robust method for noisy data^11^) as EWSRs (Fig. 5a), the proportion of populations which show EWSRs in consecutive years (Fig. 5b), and the distribution of these EWSRs prior to the recovery of populations (Fig. 5c). Fifthly, we show how the amount of training data available to the method alters the reliability of single signal and consecutive signal approaches (Fig. 6). To do this we vary the amount of data from 2 years (the minimum required to calculate a signal, see Eq. (1)) to 30 years (corresponding to including data from 2010 to the year before fishing pressures begin to decline – 2039 – in the analysis). Finally, to assess the suitability of the 2*σ* threshold proposed by Drake and Griffen^14^, we show the Receiver Operator Characteristics for a range of threshold values from 0.01 to 6*σ* for both the single and consecutive signal approaches (Fig. 7).

### ICES cod survey data

To compliment the simulation analyses described above we applied our metrics to two cod stock time series from (a) a previously overharvested stock which is thought to be slowly recovering – the North Sea, and (b) a control stock which is still being exploited and purportedly shows no signs of recovery – Western Baltic Sea (Fig. 8). To do this we combined data from two sources. Firstly, body size data on individual fish came from the ICES Database of Trawl Surveys (DATRAS), and from this individual data we calculated mean size and SD size per year over the surveyed period. Secondly, we took estimates of Spawning Stock Biomass (SSB) – which is used to assess the health of fisheries stocks – from the ICES Stock assessment database. We then assessed the biomass, mean size, and SD size data for EWSRs using the methods outlined above. In the North Sea stock, we limited our analysis to the year 1989 onwards, as this was the first year that the SSB was classified by ICES as being outside safe biological limits, and thus the analysis mimicked the analyses of the collapse period carried out in the simulation study. In the Western Baltic stocks, we used the same rationale and analyzed only data from 2001 onwards. In both cases we used half the available data for training the method (from 1989 to 2004 in the North Sea, and 2001 to 2010 in the Baltic Sea), and assessed the second half of the time series for EWSR (Fig. 8a, b).

### Reporting summary

Further information on experimental design is available in the Nature Research Reporting Summary linked to this article.

## Supplementary information

Supplementary Information

Reporting Summary

## Data Availability

All simulations were carried out using a stochastic version of the Mizer package in R^36^. Original simulation outputs are available on request from the corresponding author. ICES stock data are publicly available through the ICES data portal (http://ecosystemdata.ices.dk/). Code to carry out analyses is publicly available on https://github.com/chrit88/EWSR-Nature-Communications-2019.
