# Supplementary material for: Early warning signals of recovery in complex systems
Source: Nat Commun. 2019 Apr 11;10:1681. doi: 10.1038/s41467-019-09684-y (PMC6459826; doi:10.1038/s41467-019-09684-y)
Supplement: Supplementary file 1 — Supplementary Information [file 41467_2019_9684_MOESM1_ESM.pdf]

Supporting online information for:

Early warning signals of recovery in complex systems

Authors: Christopher F. Clements[1], Michael McCarthy[2], Julia Blanchard[3]

Affiliations:

[1]School of Biological Sciences, University of Bristol, Bristol, UK.

[2]School of BioSciences, University of Melbourne, Parkville, Melbourne, 3031, Australia.

[3]Institute for Marine and Antarctic Studies, University of Tasmania, Hobart, Tasmania, Australia.

*Correspondence to: chrisclementsresearch@gmail.com

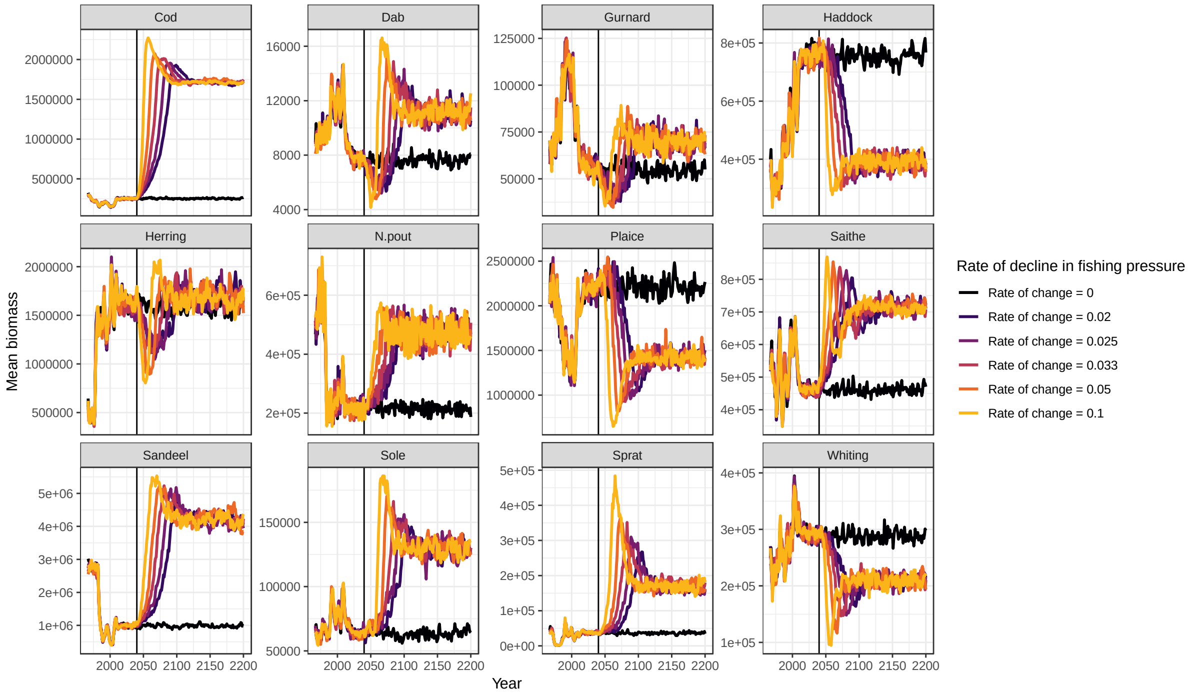

Figure S1. Mean biomass dynamics for each of the 12 species in the simulated communities under various rates of decline in fishing pressure. Each line represents the mean of 300 simulations. Vertical black lines indicate the point at which fishing pressures begin to decline (2040).

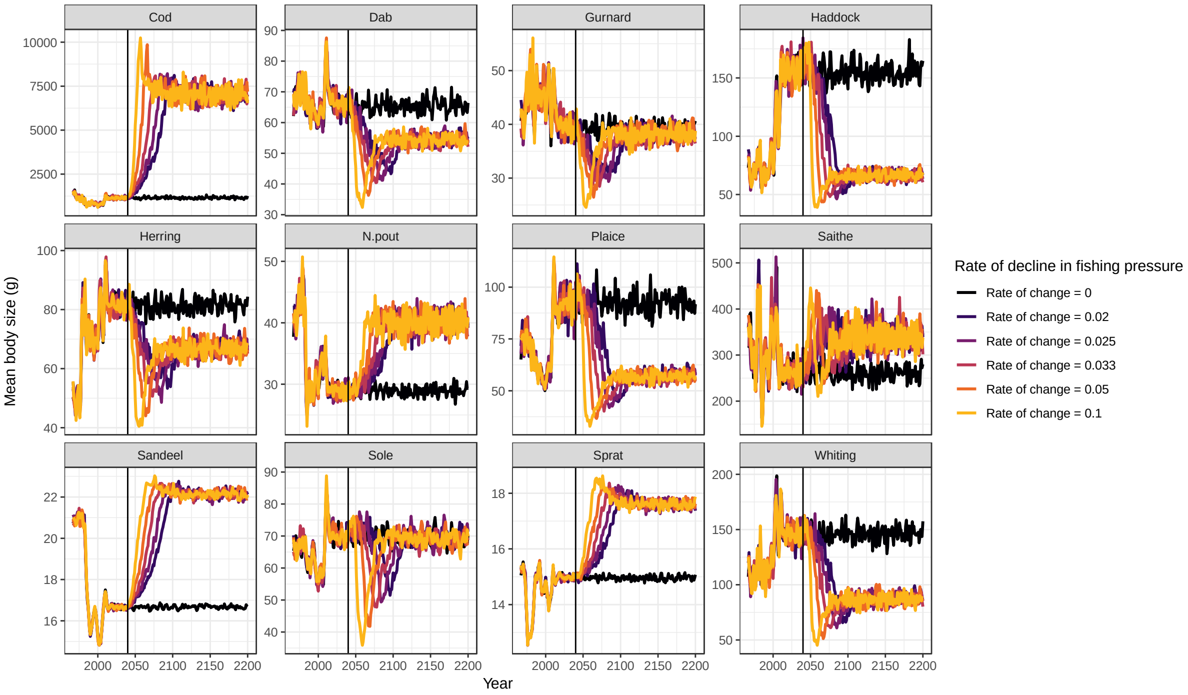

Figure S2. Mean body size dynamics for each of the 12 species in the simulated communities under various rates of decline in fishing pressure. Each line represents the mean of 300 simulations. Vertical black lines indicate the point at which fishing pressures begin to decline (2040).

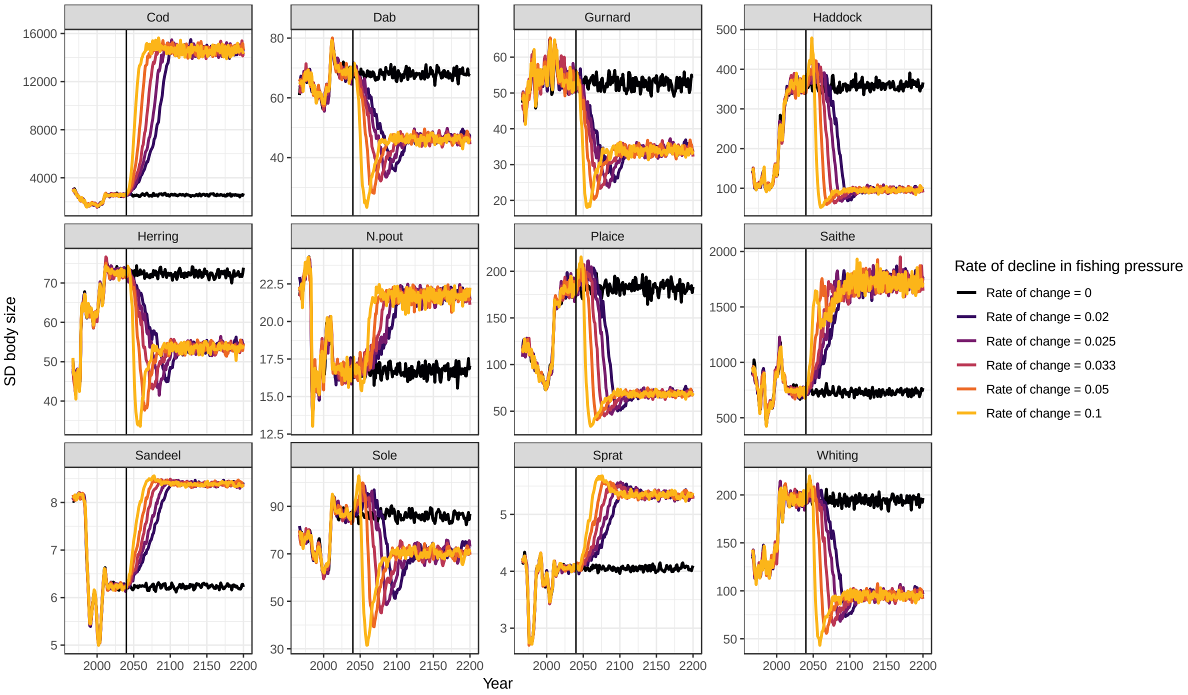

Figure S3. Mean standard deviation of body size through time for each of the 12 species in the simulated communities under various rates of decline in fishing pressure. Each line represents the mean of 300 simulations. Vertical black lines indicate the point at which fishing pressures begin to decline (2040).

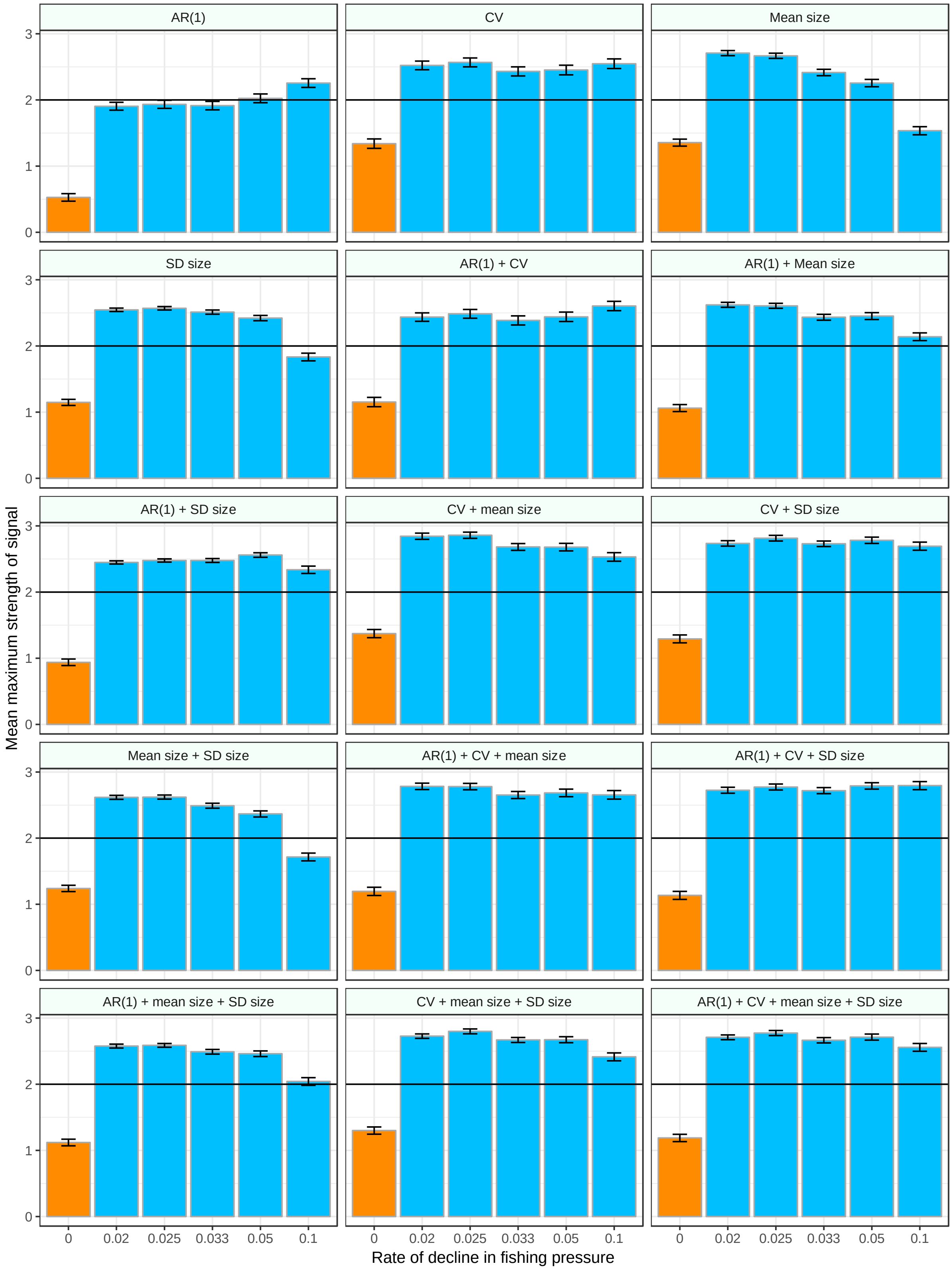

Figure S4. Maximum signal strength averaged across replicates for all of the 15 metrics tested. Black horizontal line shows the 2-sigma threshold suggested by Drake & Griffen (2010). Error bars indicate 1 standard error.

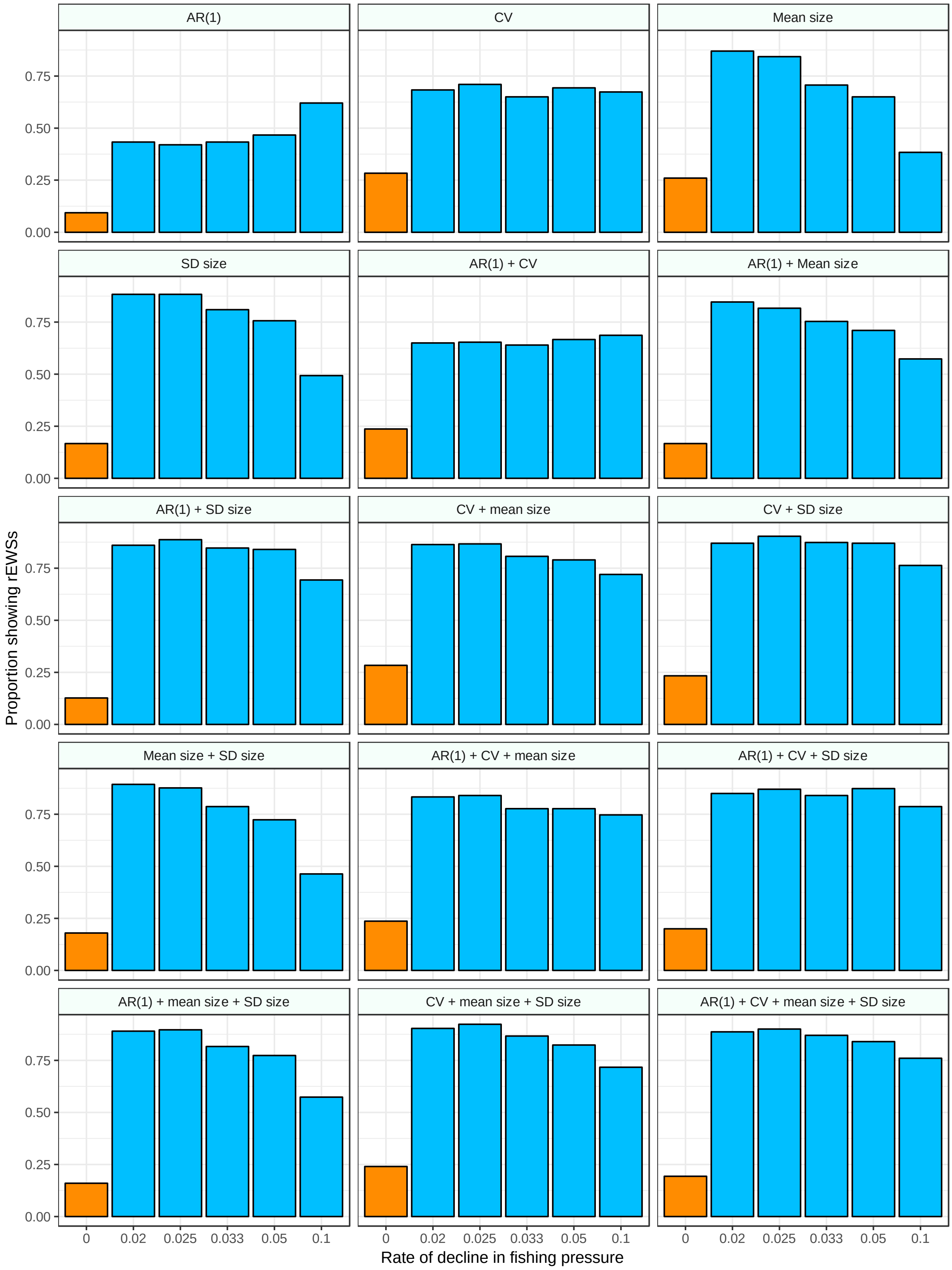

Figure S5. Proportion of time series in each rate of change of fishing pressure showing at least one EWSr at a 2 sigma threshold.

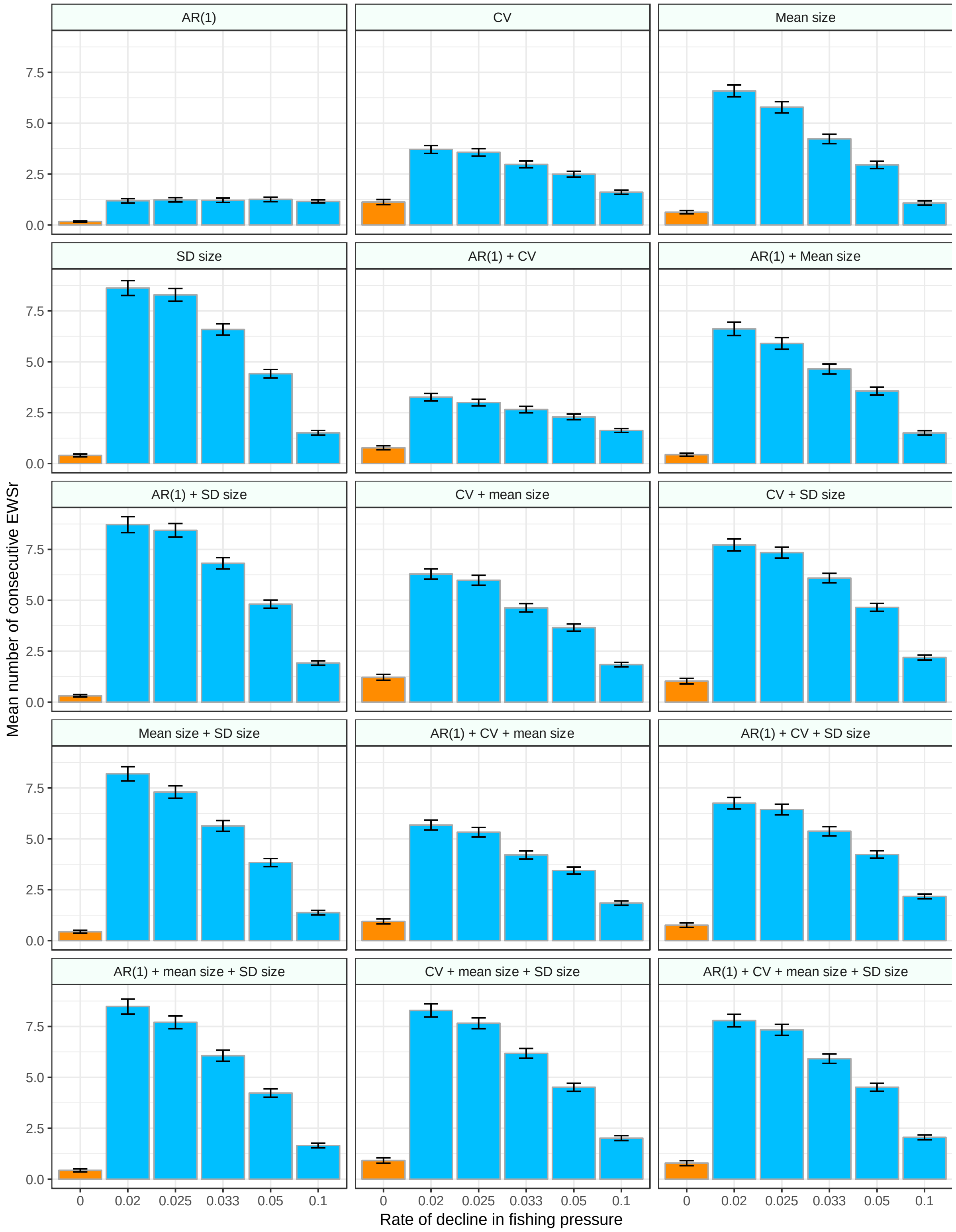

Figure S6. Mean number of concurrent signals at a 2-sigma threshold across the different fishing treatments. Error bars indicate 1 standard error.

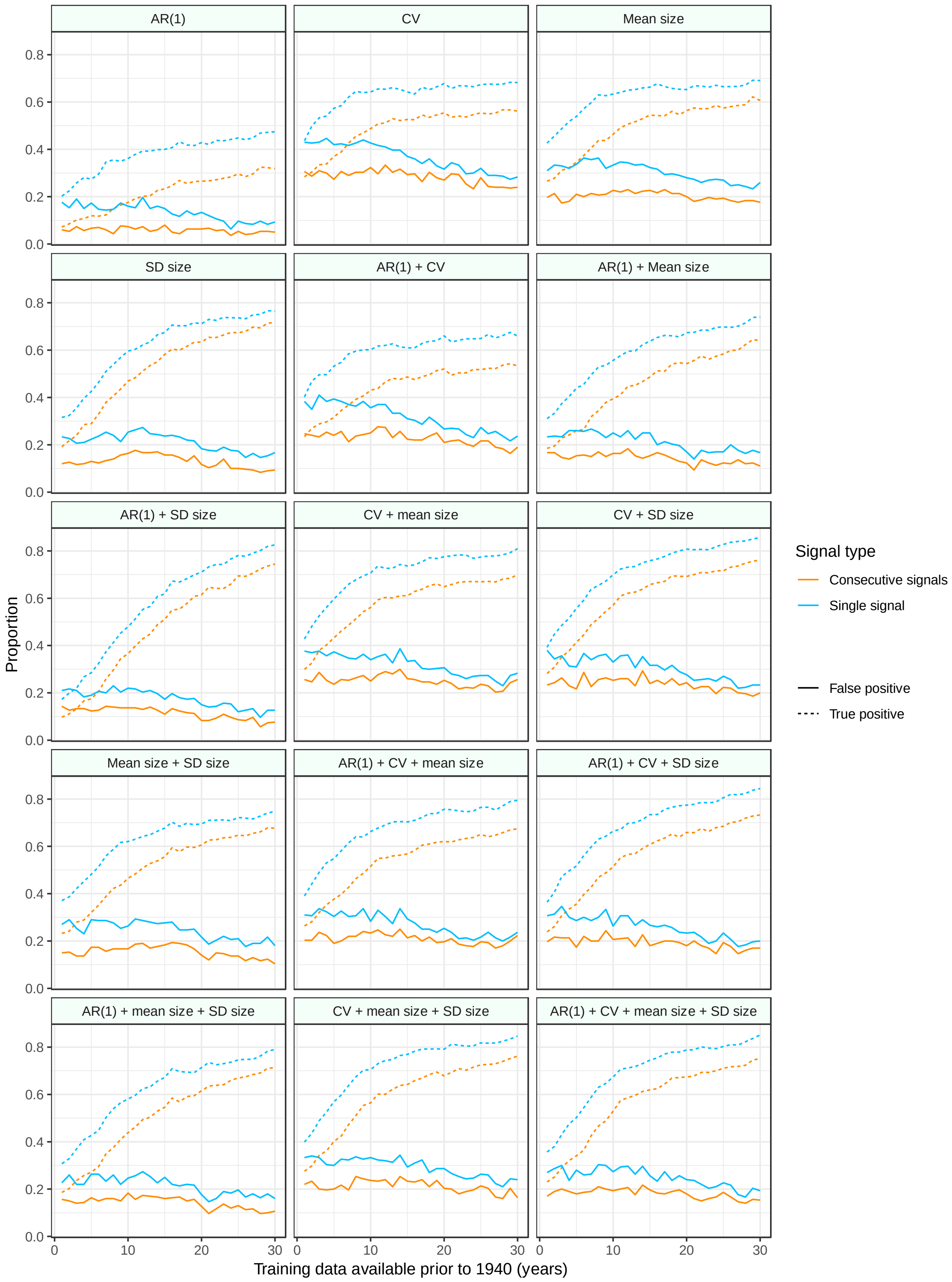

Figure S7. The effects of the amount of training data available on the proportion of false positive and true positive warning signals prior to the recovery of cod stocks across all of the metrics. Abundance-based measures (AR(1) and CV) typically performed poorly, even with 30 years of training data. Conversely those metrics which included some measure of size (either mean or SD size) typically performed well when over 15 years of training data was included in the analysis.
